# Supplementary material for: Patterns of Intron Gain and Loss in Fungi
Source: PLoS Biol. 2004 Nov 30;2(12):e422. doi: 10.1371/journal.pbio.0020422 (PMC532390; doi:10.1371/journal.pbio.0020422)
Supplement: Table S1 — Also available at http://genes.mit.edu/NielsenEtAl/. (4.3 MB ZIP). [file pbio.0020422.st001.zip › NielsenEtAl/html/342.html]

AN8293.1.NCU07011.1.MG06038.1.FG05081.1


```
 CLUSTAL W (1.82) Multiple Sequence Alignments - Introns Inserted


Sequence 1: NCU07011.1	1057 aa
Sequence 2: FG05081.1	1040 aa
Sequence 3: MG06038.1	1042 aa
Sequence 4: AN8293.1	879 aa
Alignment Length: 1067 aa
Number Identitical Residues: 351 aa
Alignment Score (without introns) 22217


MG06038.1 	MPHSQNEGPLVKRQKRNASVAQQ-QPKQPAHG-SLIFSPFR0TIGLVNSTRVPFTTIPRG
NCU07011.1	MPHSDREITSAKRQKLNASVVKSKAPQKKKKG-SSIFAPFR0TVGLVSPTSVPFTSIPLG
FG05081.1 	MPHNEADHPVVKRQKT---VAPANASKSASES-SRIFAPFR0TVGLVSPTSVPFTSIPLG
AN8293.1  	MSLTNNDGFSLPLAKRQKVVVDEAAQRNTYHAGSRIFSPFR0------------------
          	*. .: :       * .  *.     :.  ...* **:***                   

MG06038.1 	KTTFQITTCVGRCLHTYDLKRGLNLLFVTRPQTPEDITATFAHGDQVYSAWGSPRKGAKV
NCU07011.1	KTTFQITTSVGRSLQTYDLKRGLNLVFVTRPQTPADITATYAWKEKVFAAWGNPQNGE--
FG05081.1 	KTTFQISTSVGRSIQTYDLRRGLNLVFITRPQTPADITATLAWKQQIFAAWGGSTKGE--
AN8293.1  	---------------------GLKLVFISRPQTPRDITATFAWHDILFAAWGALGPGG--
          	                     **:*:*::***** ***** *  : :::***    *   

MG06038.1 	AQGIWVFQRGKKVAELELPANLNQPIHQILVFGSWIIGCAQTRIEVWKTGTLEHYTTIYT
NCU07011.1	AQGIWVYQRGKKVAELELPADLNQPIKQILIFGSWIVACAQTRLEVWKSATLEHYTTIFS
FG05081.1 	PQGVWIFQRGKKVNELVLPSDLNEPIQQIQVLGTWTIVAALTRIEIFKTATLEHYTTIHT
AN8293.1  	SGGVWVFKRGKKIASLETPIEAYAPIEHLTVFGSWIVGCNGQSVNVWKNGSYEHYTTLIL
          	. *:*:::****: .*  * :   **.:: ::*:* : .    ::::*..: *****:  

MG06038.1 	MAAAH--GGNEITGGIRNMPTYLNKIFVGRKDGWVEIWNVSTGKLIYTILPPSPKAGAVT
NCU07011.1	VAAKK--GDNELTGGVVNMPTFLNKILVGRKDGWVEIWNVSTGKLIYTILPPSPDAGSVT
FG05081.1 	TPASN--GGNEITGGIVSMPTFLNKIFVGRKDGWVEIWNVSTAKLIYTILPPGPDCGSVT
AN8293.1  	AHSRDNPGQAAYATQICNMPTYLNKVLIGKSDGTVDIWNVKTGRLVHSIFAAFPNAGPVT
          	  : ...*    :  : .***:***:::*:.** *:****.*.:*:::*:.. *..*.**

MG06038.1 	CMEPTPALSTMAIAYSSGLLMIQNILTDKPVLAVKAGTEDAP-VSTITFRTDGKGAGQAQ
NCU07011.1	CMEPTPALSLLAIAYSNGPLVIHNVLTDKASLKLMAGSEHAP-VTSISFRTDGQGAGQDG
FG05081.1 	CLEPSPALSLLAIAYSEGTLIITNVLTDKISLRIEAGSSEAQ-VKSISFRTDGMGAGSDG
AN8293.1  	VLRPSPALSVIAIAYKNGSISIQNVETGQSIFSFQPQAPRVPAITSLTFRNDDAGAGEDG
          	 :.*:**** :****..* : * *: *.:  : . . :  . ::.:::**.*. ***.  

MG06038.1 	DKDGVMATATTGSGDITFWDLNKGGRIMSVLRGAHNPPLNDGRTVRGGIGKVEFLPGQHV
NCU07011.1	TKDGVMATATSIGGDVTFWDLNKGGRVMGVLRSAHNPPSHTDKTVRGGISKVEFLPGQPV
FG05081.1 	RKDGVMATSTAATGDVTFWDLNGGGRVMGVLRSAHNPPSYEGPLVRGGISKVEFLPGQPV
AN8293.1  	QQSGAMATACLASGDITLWDLNRGGRVVGVLRNAHKVSAGDP---GSGINHIEFLDGQPV
          	 :.*.***:    **:*:**** ***::.***.**: .        .**.::*** ** *

MG06038.1 	ILTSGLDNSLKSWIFDEAALSPVPRVLHSRSGHAAPVTCLQFLPSDFDGAEAGNKWLLSG
NCU07011.1	IMTSGLDNSLKSWIFDESPFSPVPRILHMRSGHAAPVNCLQFLPTDFDGAEAGNKWLLSG
FG05081.1 	VLTSGRDNSLKSWIFDETPFSPIPRILHSRSGHAGPVNCLQFLPTDFEGADAGNKWLLSG
AN8293.1  	IVSTGKDNSLRTWIVDESPFSPIPRPLHSRSGHSDTITTVSFLPSHSDGSETTAKWLLSA
          	::::* ****::**.**:.:**:** ** ****: .:. :.***:. :*:::  *****.

MG06038.1 	GLDRSLWGWSLRRDGQSTELSQGSIRKKAKKQGILTNSALS-NGPTTTLDDLKAPEITCI
NCU07011.1	GKDRSLWGWSLRRDGQSSELSQGALRKKAKKVGILANTALGGHGPTTSLDDLKAPEITCI
FG05081.1 	GQDRSLWGWSLRRDGQSAELSQGNIRKKAKKIGILATNALS-HGPTTTLEDLKAPEITCM
AN8293.1  	SKDSSLWGFSLRKDSQSTELSQGSVEQGAKKKGVVISDVNN--GSISSQPNLRAPEVTCI
          	. * ****:***:*.**:***** :.: *** *:: . . .  *. ::  :*:***:**:

MG06038.1 	ASSLNRDGGIGALPGKQRIWAN--QQGKNVDAEVSGMTGWESVITAHRNDPKARTWFWGR
NCU07011.1	ASSLNRDGGMGAMPGRQVIWDKGNDKSKPTKSELAAMTGWESVVTAHKNDPWARTWFWGR
FG05081.1 	ASSLNRDGGMGAMPGNQPIWQK-SQKKRAHNAEVSGMTGWESVVTAHKNDSYARTWFWGR
AN8293.1  	ACSLNRDGGMGVTVSG-TIWSN----PKTTDTTASNKTGWESIVTGHRGDKYARTWFWGK
          	*.*******:*.  .   ** :     :  .:  :  *****::*.*:.*  *******:

MG06038.1 	KKAGRWAFPTGDGSYVS~TVAISPCG~TFALVGSMEGGVDMYNLQSGQHRQRFPSKLTPA
NCU07011.1	KRAGRWAFKTGDGENVS~TVAISPCG~SFALVGSVGGSLDMFNLQSGRHKQRFPSRLTPA
FG05081.1 	KRAGRWAFPTGDDTNVS~TVAISPCG~TFALVGAESGGIDMYNLQSGNHRQRFPSRLTPA
AN8293.1  	KKAGRWVFETGDGTDVK0SVAISQCG1--------------------QYRRSYPCQKTKY
          	*:****.* ***.  *. :**** **                     .::: :*.: *  

MG06038.1 	QARQLKVQQLRQANSELMQLEYNSK--STFLPGSGRHKKAVTGIVVDSMLKHVITCSLDG
NCU07011.1	QMRQLKMQQLKALD-KVSQLEKRASSKTSFAPGSGKHTKSVTGIVVDPLNRFVISCSLDG
FG05081.1 	QARQLKMQQLRQAD-DVVQLHMEST--RQFPIGTGKHTKRITGLVVDSMNKTVVSCSLDG
AN8293.1  	DLGGEVRYSNKQIH--------------------TQHTKSVTGLAIDSLNRTVLSCGLDG
          	:       . :  .                     :*.* :**:.:*.: : *::*.***

MG06038.1 	TVK~FWDFLTGALLDQIDWAPMTSIIGCRYHSGSNLLAFSCDDNSIRVVDMETKRTIREF
NCU07011.1	TVK~FWDFVTGNLVDEIDWAPMVQITGCRYHAGNDLIAFACDDNSIRVVDIETKQTIREF
FG05081.1 	KIK~FWDFLTGNLLEQIDWAPMTFPTGCRYHPASNLLAFSCDDLSIRVVDMETKRTIREF
AN8293.1  	KVK0FWDFQSGLLIKELDWYPMTAITALRYNSMSELVAFSCDDLSIRVVDMETKKIVREL
          	.:* **** :* *:.::** **.   . **:. .:*:**:*** ******:***: :**:

MG06038.1 	WGCQSTINDFCFSPDGRWIVAASQDSTLRVWDLPTSHLIDAVRLEQPCKAVSFSSTGEFL
NCU07011.1	WGCRDTINDFTFSNDGRWIVAASQDRVVRVWDLPTAHLIDAFRLEKPCTAIAFSNTGEYL
FG05081.1 	WGPQGAINDFCFSSDGRWIIAASQDCVIRVWDLPTSHMIDAIRLEKPCKAIAMSVTGDYL
AN8293.1  	WGCAGQINDFIFSSDGRWIIAASMDSIIRVWDLPTGHLIDLFRVSSTCTSLAMSPTGDFL
          	**  . **** ** *****:*** *  :*******.*:** .*:...*.::::* **::*

MG06038.1 	ATATEGELGVHLWTNRTLFKHVATKQISEKEIAHISA-PTTSGEGGFGIVDAAFEDDAPE
NCU07011.1	AGATEGELGVHIWTNRTLFRHVPTRQISEKDIAEISAGPTSSGEGGQGLIEAAFEDEQED
FG05081.1 	AATIENELGVTLWTNKALFKHVPTRQISEKEIGHVSA-PTVSGENSNGMLEGAFEGEQAE
AN8293.1  	ATAHADGVGIGLWSNRSLFIPVSTRDLNVDNLLDTGL-PTSSGEGGVGIIEAAFQQDNEE
          	* :  . :*: :*:*::**  *.*:::. .:: . .  ** ***.. *:::.**: :  :

MG06038.1 	DND-IITAPSLDQLSADLMTLSLVPKSKWQTLLHLDLIK~NRNKPKEAPKVPEKAPFFLP
NCU07011.1	EEDEGVSAPVLDQLSADMVTLSLVPKSRWQTLLHIDLIK~QRNKPKEPPKAPEKAPFFLP
FG05081.1 	DED-TVVAPTIEQLSADLTTLSLVPKSRWQTLLHLDLIK~ERNKPKEAPKAPEKAPFFLP
AN8293.1  	HDG-DGPISCTEQLDRDMLTLSVVPKNKWQTLVHMDLIK0ERNMPKEPPKRPEKAPFFLP
          	.:.     .  :**. *: ***:***.:****:*:**** :** ***.** *********

MG06038.1 	PVGEQNS----IDAVAKTDKTSDSQSRITALDMSR---SQEAFTAKLRAGAASGNY1DGF
NCU07011.1	SLRDGTTNPLLLTEGEKKDEEESRISKLTQLETTR---TYQTFTAKLLEGAETGDY1TPF
FG05081.1 	STTGKDT---LMANKEQKGDIEN-TSRVTKLEQAR---FEQALSSKLRVGAETGNY1DEF
AN8293.1  	APASDGAPKHNANATLLEEGKLTESSRVAKLQITKNPNAVSRFTSLLRTGCISGDF~EPF
          	.     :                  *::: *: ::...  . ::: *  *. :*::   *

MG06038.1 	IEHLKSLSPSTADLELRSLSSS------DGDFNELLHFIKALTARLVARRDYELMQAWMT
NCU07011.1	ITHLKSLSPSTADLELRSLSIGGLDPESSEDSNELLHFIRAMTQRLAQRRDYELTQAWMT
FG05081.1 	IEHLKSLSPSSADLELRSLSIG----DGDDVPNELLHFIRALTARLKTRRDYELTQAWMT
AN8293.1  	FFEMKSLNPS----------------------------------RLDFRHDR--------
          	: .:***.**                                  **  *:*         

MG06038.1 	VFLRLHCDQVVLDDVLIEALRQWKVQEEKEKERLDNLVGYCGGVVGFLRSPLT
NCU07011.1	VFLRLHFDVIMESETLLKELGKWKEQQEREKKRLDDLVGYCGGVVGFLRSPRT
FG05081.1 	VFLRLHFDLVMENQDLLEALSEWKTYQEKECTRLDGLVGYCSGVVSFLRSPRT
AN8293.1  	-----------------------------------------------------
          	
```
